# Robust plan optimization using edge-enhanced intensity for intrafraction organ deformation in prostate intensity-modulated radiation therapy

**DOI:** 10.1371/journal.pone.0173643

**Published:** 2017-03-10

**Authors:** Iori Sumida, Hajime Yamaguchi, Indra J. Das, Yusuke Anetai, Hisao Kizaki, Keiko Aboshi, Mari Tsujii, Yuji Yamada, Keisuke Tamari, Yuji Seo, Fumiaki Isohashi, Yasuo Yoshioka, Kazuhiko Ogawa

**Affiliations:** 1 Department of Radiation Oncology, Osaka University Graduate School of Medicine, Suita, Osaka, Japan; 2 Department of Radiation Oncology, NTT West Osaka hospital, Tennoji-ku, Osaka, Japan; 3 Department of Radiation Oncology, New York University Langone Medical Center, New York, New York, United States of America; North Shore Long Island Jewish Health System, UNITED STATES

## Abstract

This study evaluated a method for prostate intensity-modulated radiation therapy (IMRT) based on edge-enhanced (EE) intensity in the presence of intrafraction organ deformation using the data of 37 patients treated with step-and-shoot IMRT. On the assumption that the patient setup error was already accounted for by image guidance, only organ deformation over the treatment course was considered. Once the clinical target volume (CTV), rectum, and bladder were delineated and assigned dose constraints for dose optimization, each voxel in the CTV derived from the DICOM RT-dose grid could have a stochastic dose from the different voxel location according to the probability density function as an organ deformation. The stochastic dose for the CTV was calculated as the mean dose at the location through changing the voxel location randomly 1000 times. In the EE approach, the underdose region in the CTV was delineated and optimized with higher dose constraints that resulted in an edge-enhanced intensity beam to the CTV. This was compared to a planning target volume (PTV) margin (PM) approach in which a CTV to PTV margin equivalent to the magnitude of organ deformation was added to obtain an optimized dose distribution. The total monitor units, number of segments, and conformity index were compared between the two approaches, and the dose based on the organ deformation of the CTV, rectum, and bladder was evaluated. The total monitor units, number of segments, and conformity index were significantly lower with the EE approach than with the PM approach, while maintaining the dose coverage to the CTV with organ deformation. The dose to the rectum and bladder were significantly reduced in the EE approach compared with the PM approach. We conclude that the EE approach is superior to the PM with regard to intrafraction organ deformation.

## Introduction

Intensity-modulated radiation therapy (IMRT) and volumetric modulated arc therapy are commonly used for the treatment of prostate cancer due to their superior dose conformality to the target and sparing of normal tissues such as the rectum and bladder when compared to three-dimensional (3D) conformal radiotherapy. To create the planning target volume (PTV), the clinical target volume (CTV) delineated by a physician is expanded to incorporate a setup margin based on the setup error and an internal margin for intrafraction organ motion [1]. Treatment planners take account of the dose constraints in both the target and normal tissues to ensure a satisfactory dose distribution and clinically relevant dose–volume indices. However, even when this is achieved successfully, it may not always be optimal, as described by Roy et al. [2]. Even when the required indices can be satisfied within the predefined constraints, it is preferable to keep the dose to normal tissues as low as possible.

Several approaches have been proposed for minimizing the dose to normal tissues while ensuring the prescribed dose coverage for the target; these include the individualized margin [3], anisotropic margin [4, 5], and zero-PTV-margin [6]. These margin approaches take into consideration the interfraction motion at setup as well as intrafraction organ motion during the irradiation. Organ motion can blur the dose distribution at the beam edge [7], resulting in an underdose in peripheral regions of the target, and so another technique has been proposed using edge-enhanced (EE) intensity maps with a combination of margins [8]. Either the margin or the EE technique can be used to address the underdose and compensate the dose distribution.

For dose evaluation regions with relatively homogeneous density, such as the pelvic region, the static dose cloud approximation described by Craig et al. [9] may be useful for estimating the dose distribution, including interfraction and intrafraction organ motion. This approximation makes it possible to estimate the dose under organ motion and deformation by considering the organ elements (voxels) to be moving within a static dose cloud distribution [10–11]. The organ motion is represented by a probability density function (PDF) applied to the voxel elements in each organ [12].

Trofimov et al. [13] have presented a stochastic “motion kernel” approach for 4D IMRT optimization for lung and liver cancer to compensate for the dose-blurring effect due to organ motion. The target trajectory in the superior–inferior (SI) direction is obtained using 4D computed tomography (CT) datasets and used to create the PDF, which is applied to all parts of the patient’s anatomy [13]. The authors concluded that this approach did not provide a dosimetric advantage compared to tracking or gating approaches, but that it could result in more efficient delivery.

In prostate cancer treatment, random variations of the rectum [14] and bladder [15] during radiotherapy have been reported, with variable motion pattern and size. Intrafraction organ motion has been investigated for the evaluation of dose distribution to the prostate, and there have been a few reports about intrafraction tumor deformation in which a tumor deformation tracking system was achieved via a dynamic multileaf collimator approach [16]. In the present study, we applied a different approach using an EE technique in the treatment planning process to account for intrafraction tumor deformation. This study was based on a simulation of the dose distribution calculated by the treatment planning system (TPS), focusing on the dosimetric effect in the presence of random organ deformation without the patient setup error. Using this approach, the PDF could be applied to each voxel in each organ. The purpose of this study was to create a treatment plan for prostate IMRT using an EE intensity map to address the dose-blurring effect caused by organ deformation, and to compare this approach with the commonly used PTV-margin (PM) approach.

## Materials and methods

The analysis included the data for 37 prostate cancer patients who were treated with fixed-gantry step-and-shoot IMRT using a 10-mm multileaf collimator leaf width. This retrospective study was approved by the local ethics committee at NTT West Osaka hospital. All participants provide their written informed consent to participate in this study, and their analyzed data are anonymized. A beam energy of 10 MV was generated by an X-ray machine equipped with a linear accelerator (ONCOR Impression Plus; Siemens Medical Systems, Concord, CA). CT images with a slice thickness of 2.5 mm were acquired under free breathing with a VacLok fixation cushion (CIVCO Medical Solutions, Orange City, IA) using a GE BrightSpeed CT scanner (GE Medical Systems, Milwaukee, WI). A CTV including the prostate gland and proximal seminal vesicle was delineated by a radiation oncologist. After the rectum and bladder were delineated as normal tissue, a medical physicist optimized the dose distribution using a XiO treatment planning system (Elekta Instrument AB, Stockholm, Sweden). The prescription dose was 78 Gy in 39 fractions to the CTV D_95%_ (the dose received by 95% of the volume of the CTV). The dose constraints for the rectum and bladder were adapted from reference [17], in which the percentage volumes receiving 65 Gy and 40 Gy (V_65Gy_ and V_40Gy_) in the rectum should be less than 17% and 35%, respectively, and in the bladder less than 25% and 50%, respectively.

### Creation of the de-blurring dose distribution

**Fig 1:**
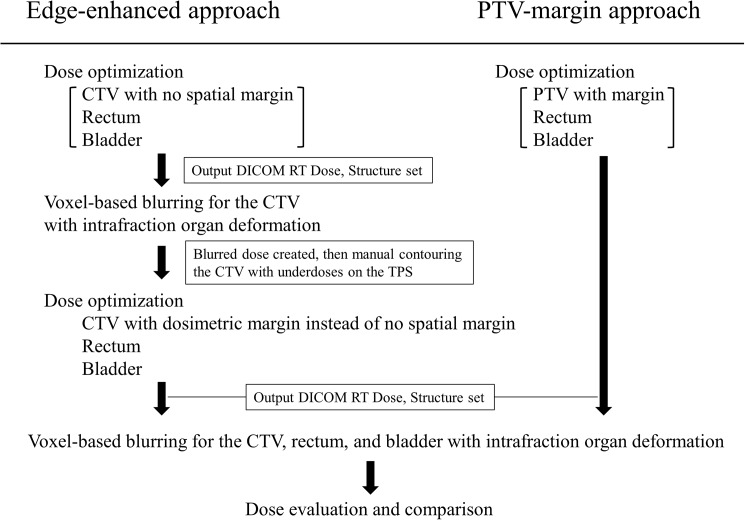
The treatment planning process for dose evaluation as applied in the edge-enhanced and planning target volume (PTV)-margin approaches. For the edge-enhanced approach, dose optimization to the clinical target volume (CTV) with no spatial margin was applied, followed by the creation of blurred dose distribution. The underdose region within the CTV was calculated, and the manual delineation was performed on the TPS according to the region. Re-optimization was performed to create the edge-enhanced dose distribution. In contrast, for the PTV-margin approach, dose optimization to the PTV with margin was applied. Finally, the two planned dose distributions derived from the edge-enhanced and PTV-margin approaches were blurred and compared for evaluation.

**Fig 2:**
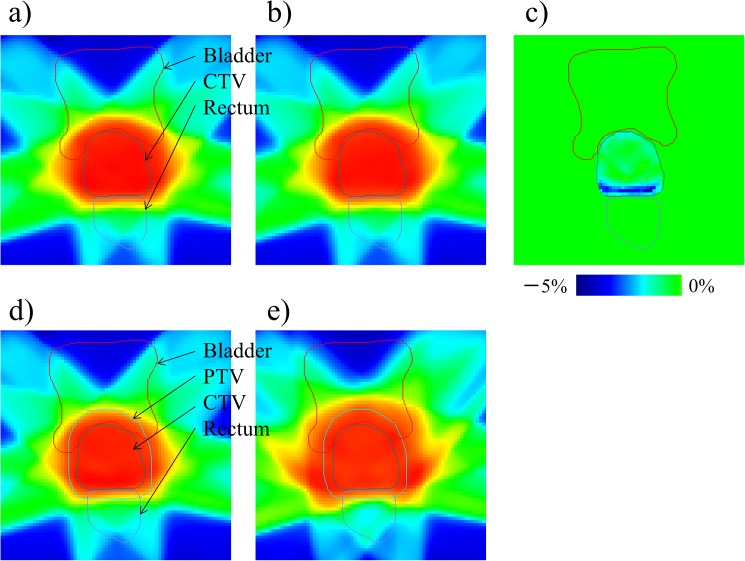
**The dose distributions: a) optimized for the clinical target volume (CTV) with no internal margin before applying the edge-enhanced approach; b) blurred only to CTV derived from a); c) the subtraction image derived from a) and b); d) the edge-enhanced approach created by re-optimization with 5% scaled up the lower dose constraint to the CTV via simultaneous integrated boost technique; and e) the planning target volume (PTV)-margin approach created by optimization to the PTV with margin. In c), the color bar represents the range of 5% dose difference**.

It was assumed in this study that the patient setup error would be perfectly corrected in each treatment fraction by image guidance in both EE and PM approaches, which resulted in the translation errors of zero, and thus, no setup margin was used; we, therefore, considered only the intrafraction organ deformation for the treatment plan over the treatment fractions. The treatment planning processes following the EE and PM approaches are presented in Fig 1. First, for the EE approach, the optimized dose distribution to the CTV with no internal margin and to the rectum and bladder without intrafraction organ deformation was necessary before creation of edge-enhanced intensity. The dose constraints were in accordance with the dose constraints specified in the previous section. The optimized dose distribution is shown in Fig 2A. Next, the DICOM RT files including the plan, structure set, and dose were then exported. The resolution of the dose grid was 2 mm. The dose distribution at this time point is referred to as the static dose distribution (D_stat_). These files were imported into our in-house software developed using Delphi2007 (Borland Software Corporation, Austin, TX), which can create a probability dose distribution to each voxel in each organ based on the motion PDF. It was assumed that the motion PDF has three components, including axes of motion in the left–right (LR), anterior–posterior (AP), and superior–inferior (SI) directions, and the motion probability was based on the normal distribution with a standard deviation. The magnitudes of intrafraction motion for the CTV [18], rectum [14], and bladder [15] in the three axes (SI, LR, and AP) were used in the reports in the respective references and are summarized in Table 1. The values given for the CTV and rectum are two standard deviations, and those for the bladder are maximums. Because of the central limit theorem [19], even when motion probability presents a non-uniform distribution, after many fractions the ultimate probability distribution will converge towards a Gaussian shape. Although the presented deviations are derived from the intrafraction organ motion for the organs, in this study these values were referred to as, and assumed to be, the magnitude of the organ deformation.

**Table 1:** Magnitudes of intrafraction organ deformation in the three directions for the clinical target volume (CTV), rectum, and bladder.

Organ	LR (mm)	AP (mm)	SI (mm)	Reference
CTV	6	8	6	[17]
Rectum	6	10	0	[13]
Bladder	5	9	10	[14]

Values denote two standard deviations (for CTV and rectum) or maximum (for bladder). LR: left–right; AP: anterior–posterior; SI: superior–inferior.

In order to calculate the stochastic dose for the CTV (D_stoc_) in the presence of intrafraction organ deformation, the location of the dose grid in D_stat_ was changed 1000 times [20] to fulfill the normal distribution criterion as expressed in Eq (1).

$$D_{stoc}(x,y,z)=\sum_{i=1}^{1000}D_{stat}(x+\sigma_{i}^{LR},y+\sigma_{i}^{AP},z+\sigma_{i}^{SI})/1000,$$ (1)

where D_stoc_(*x*, *y*, *z*) is the mean value of the stochastic dose at location (*x*, *y*, *z*), which is the same as the DICOM RT-dose grid, and $\sigma_{i}^{LR}$, $\sigma_{i}^{AP}$, and $\sigma_{i}^{SI}$ are the probable *i*-th shifts in location in the LR, AP, and SI directions, respectively, with the locations changed 1000 times randomly (based on the normal distribution). The coordinates *x*, *y*, and *z* refer to the LR, AP, and SI directions, respectively. Because the probable shift location is not identical to the dose grid location, 3D linear interpolation was applied to calculate the stochastic dose that resulted in the blurred dose for the CTV shown in Fig 2B.

Based on the blurred dose distribution D_stoc_ for the CTV, the underdose region (shown in Fig 2C) calculated by the subtraction of two dose distributions (D_stoc_–D_stat_) that received a dose 5% lower than the prescribed dose was delineated manually in the TPS. The dose was then re-optimized to create the EE dose distribution shown in Fig 2D for that region in the CTV accompanied the 5% scaled up of the lower dose constraint, which is the same as that by the simultaneous integrated boost technique [21], indicating that the dosimetric margin of 5% scaled up of the lower dose constraint was applied to the CTV instead of the spatial margin, i.e., internal margins. The dose constraints were not changed for the rectum and bladder. The optimized EE dose shown in Fig 2D is referred to as D_edge_.

### Dose under intrafraction organ deformation

Under the EE dose delivery approach, we evaluated the dosimetric effect for the CTV, rectum, and bladder that resulted from the intrafraction organ deformation. The motion PDF based on the magnitudes of motion presented in Table 1 was applied to each dose grid in the organ using Eq (1). To allow a comparison with the PM technique (Fig 1), the PTV was created in the TPS by adding margins of 6, 8, and 6 mm in the LR, AP, and SI directions, respectively. Again, the size of these margins was 2 SD of the random prostate motion, with no setup error [18]. The same target dose constraint was used for the PTV but not for the CTV; again, the dose constraints were not changed for the rectum and bladder. The prescription dose was consistent with the specified 78 Gy in 39 fractions to the CTV D_95%_. Finally, the dose distribution (D_PM_) was optimized (Fig 2E).

We investigated the following two questions, which focused on the CTV and the normal tissues. With the EE dose delivered under intrafraction organ deformation to the CTV, would the dosimetric parameters described earlier be comparable with those for the PM approach? With the EE dose delivered under intrafraction organ deformation to the normal tissues, how would the high-dose region derived from the EE approach deliver to the rectum and bladder, and would the doses be increased or decreased compared with those of the PM approach? The anterior side of the rectum and the inferior side of the bladder are close to the CTV.

Initially, the total monitor units and the number of segments were compared between the planned doses D_edge_ and D_PM_. The blurred doses D_edge_^blur^ and D_PM_^blur^ that accompanied the intrafraction organ deformation were then simulated using the following Eqs (2) and (3). The probable shift amount was the same as in Eq (1) and referred to in Table 1.

$$D_{edge}^{blur}(x,y,z)=\sum_{i=1}^{1000}D_{edge}(x+\sigma_{i}^{LR},y+\sigma_{i}^{AP},z+\sigma_{i}^{SI})/1000,$$ (2)

$$D_{PM}^{blur}(x,y,z)=\sum_{i=1}^{1000}D_{PM}(x+\sigma_{i}^{LR},y+\sigma_{i}^{AP},z+\sigma_{i}^{SI})/1000.$$ (3)

The following dose–volume indices were calculated for the comparisons of D_edge_^blur^ and D_PM_^blur^: the generalized equivalent uniform dose (gEUD) in Gy, the homogeneity index (HI), and the conformity index (CI) for the CTV. HI is defined as (D_2%_ − D_98%_)/D_50%_, as discussed in ICRU Report 83 [22]. The CI is defined in ICRU Report 50 [1] as the ratio of the volume receiving the prescribed dose to the volume of the PTV. In this study, we evaluated the CI for the CTV instead of the PTV because of no PTV in the evaluated dose for D_edge_. Thus, the CI was calculated for the planned doses D_edge_ and D_PM_. The gEUD, V_65Gy_, and V_40Gy_ were calculated for the rectum and bladder. The gEUD was calculated for each organ using Niemierko’s phenomenological formula [23] given by the following equation:

$$gEUD={\left(\frac{1}{N}\sum_{i=1}^{N}D_{i}^{a}\right)}^{\frac{1}{a}},$$ (4)

where *N* is the number of voxels in each organ and *a* is the tumor- or normal tissue-specific dose–volume effect parameter. For radiobiological evaluation, the tumor control probability (TCP) was calculated using Niemierko’s EUD-based model [24] given by the following equation:

$$TCP=\frac{1}{1+{\left(\frac{{TCD}_{50}}{EUD}\right)}^{4\gamma 50}},$$ (5)

where EUD is the gEUD for the CTV, TCD_50_ is the dose required to achieve 50% TCP, and γ50 is the normalized tumor dose–response slope at TCD_50_. The individual voxel NTCP (P) was calculated using the relative seriality model [25] given by the following equation:

$$P(D_{i})=2^{-e^{e\gamma 50(1-\frac{D_{i}}{D_{50}})}},$$ (6)

where D_i_ is the dose delivered to the *i*-th voxel, D_50_ is the dose required to achieve 50% NTCP, and γ50 is the normalized normal tissue dose–response slope at D_50_. The following equation was used to calculate NTCP for the organ, incorporating the functional architecture of relative seriality:

$$P={\left[1-\prod_{1}^{n}{\left[1-P{\left(D_{i}\right)}^{s}\right]}^{\Delta v_{i}}\right]}^{\frac{1}{s}},$$ (7)

where *n* is the number of sub-volumes in the organ, *s* is the relative seriality factor, which ranges between 1 for serial organs and 0 for parallel organs, and Δ*v*_i_ is defined as *v*_i_/*V*, where *v*_i_ denotes the sub-volume in the differential dose–volume histogram and *V* denotes the total volume of the organ. These parameters were used to calculate TCP and NTCP with the endpoints in each structure summarized in Table 2.

**Table 2:** Radiobiological parameters used to calculate the generalized equivalent uniformed dose, tumor control probability, and normal tissue complication probability.

Structure	Endpoint	Tissue-specific parameter *a*	TCD_50_/D_50_ (Gy)	γ50	s	Study (Ref)
CTV	Local control	−13	67.5	2.2		[26]
Rectum	Grade 2 rectal bleeding	8.33	83.1	1.69	0.49	[27]
Bladder	Symptomatic contracture	2	80	3	0.18	[28]

Abbreviations: TCD_50_: dose required for 50% probability of tumor control; D_50_: dose at which there is a 50% probability of normal tissue complication; γ50: slope at the TCD_50_ or D_50_; s: relative seriality factor; CTV: clinical target volume.

### Statistical analysis

The normality of the data was checked with the Shapiro–Wilk test, and comparison analyses used the two-tailed paired *t*-test or the Wilcoxon signed-rank test as appropriate. A *p*-value less than 0.05 was considered statistically significant. Statistical analyses were performed using R version 3.1.2 statistical software (R Foundation, Vienna, Austria).

## Results and discussion

Comparisons of the total monitor units, the number of segments, and the CI values are summarized in Table 3. The total monitor units with the EE approach were significantly lower than with the PM approach (*p* < 0.001). The number of segments was also significantly smaller (*p* < 0.001). The CI for the EE approach was as low as 44% of that for the PM approach (*p* < 0.001). This indicates the volume irradiated by the prescribed dose was widely spread over the CTV in the PM approach.

**Table 3:** Comparison of total monitor units, the number of segments, and the conformity index in the plans created using the edge-enhanced approach and PTV-margin approach for 37 prostate cancer patients.

Index	Edge-enhanced (EE) approach	PTV-margin (PM) approach	*p*-value
Total monitor units	412.1 ± 49.7	515.9 ± 88.8	<0.001
The number of segments	53 ± 13	77 ± 19	<0.001
Conformity index	1.70 ± 0.19	3.04 ± 0.39	<0.001

Values are means ± SD.

The dose–volume parameters and radiobiological indices in the CTV, rectum, and bladder for the EE and PM approaches are summarized in Table 4. These were calculated for D_edge_^blur^ and D_PM_^blur^. For the CTV, gEUD with the EE approach was 79.26 Gy, which was significantly smaller than that with the PM approach (*p* < 0.02). A subtle difference (of less than 0.3 Gy) was observed between the EE and PM approaches. Accordingly, the TCP with the EE approach was significantly smaller than that with the PM approach (*p* < 0.02). The HI with the EE approach was 0.06, which was significantly larger than that with the PM approach (*p* < 0.001).

**Table 4:** Dose–volume parameters and radiobiological indices for the edge-enhanced approach and PTV-margin approach under intrafraction organ deformation.

Index	Edge-enhanced (EE) approach	PTV-margin (PM) approach	*p*-value
CTV gEUD (Gy)	79.26 ± 0.28	79.50 ± 0.50	<0.02
CTV TCP	0.8043 ± 0.0049	0.8082 ± 0.0084	<0.02
CTV HI	0.06 ± 0.01	0.05 ± 0.02	<0.001
Rectum V_65Gy_ (%)	4.27 ± 1.34	5.34 ± 1.41	<0.001
Rectum V_40Gy_ (%)	28.46 ± 2.51	30.30 ± 3.33	<0.001
Rectum gEUD (Gy)	51.09 ± 1.37	52.19 ± 1.48	<0.001
Rectum NTCP	0.0045 ± 0.0011	0.0058 ± 0.0015	<0.001
Bladder V_65Gy_ (%)	8.24 ± 3.65	17.21 ± 5.17	<0.001
Bladder V_40Gy_ (%)	25.29 ± 9.28	37.57 ± 9.28	<0.001
Bladder gEUD (Gy)	33.25 ± 6.36	41.48 ± 5.30	<0.001
Bladder NTCP	0.0001 ± 0.0001	0.0013 ± 0.0016	<0.001

Values are means ± SD (*N* = 37). gEUD: generalized equivalent uniform dose; TCP: tumor control probability; NTCP: normal tissue complication probability; HI: homogeneity index; V_65Gy_: organ volume receiving 65 Gy; V_40Gy_: organ volume receiving 40 Gy. Values are means ± SD.

For the normal tissues, V_65Gy_ and V_40Gy_ for both approaches achieved the predefined dose constraints. For the rectum, V_65Gy_ and V_40Gy_ with the EE approach were significantly smaller than those with the PM approach (*p* < 0.001). The gEUD with the EE approach was significantly smaller than that with the PM approach (*p* < 0.001). Accordingly, the NTCP value with the EE approach was significantly smaller than that with the PM approach (*p* < 0.001). For the bladder, V_65Gy_ with the EE approach was 8.24%, which was one-half of that with the PM approach (*p* < 0.001). V_40Gy_ with the EE approach was 12% smaller than that with the PM approach (*p* < 0.001). The gEUD with the EE approach was 8 Gy smaller than that with the PM approach (*p* < 0.001). Accordingly, the NTCP value with the EE approach was significantly smaller than that with the PM approach (*p* < 0.001).

The EE approach was applied to the CTV to ensure the treatment delivery was robust, and the dosimetric impact was evaluated in the presence of intrafraction organ deformation for both the EE and PM approaches.

The number of segments and total monitor units with the EE approach were remarkably reduced compared with the PM approach. Because the EE approach did not use a spatial margin for the CTV but instead used the dosimetric margin, this meant that the beam intensity was increased at the edge of the CTV. Compared with the PM approach, the irradiated area in each beam (based on the beam’s eye view) resulted in a small region limited in the CTV. This would explain why the CI was lower with the EE approach than with the PM approach.

For the size of organ deformation, we referred to intrafraction organ motion data from published reports [14, 15, 18] and applied the same size into each dose grid in each organ to estimate the blurred dose distribution stochastically. In actual treatment, the intrafraction organ motion does not always correspond with the organ deformation, and the deformation size would vary in part of the organ [29], which may be a limitation of this study. Some investigators have reported that the sizes of intrafraction organ motion for the prostate and seminal vesicle are measured by 4D MRI acquisition with the assumption of the treatment time [30, 31, 32] and have concluded that the determination of appropriate intrafraction margins to accommodate the time-dependent uncertainty in positional targeting is a topic for the on-line image guidance model [32]. If the size of intrafraction organ motion for those organs could be calculated as the size of organ deformation by a deformable image registration technique, the blurred dose might be estimated more accurately.

The dose distribution inside the CTV (except for the peripheral region) was homogeneously optimized in the treatment planning process; thus, even the dose was blurred with the stochastic approach using Eq (1), and the dose did not differ greatly from the planned dose. In contrast, the voxels in the peripheral region of the CTV received an underdose by the blurring process, as shown in Fig 2C. The underdose region and its effect on the dose would depend on how the treatment plan was optimized based on the dose constraints of the organs. When there was a tighter dose gradient at the interface between the posterior side of the CTV and the anterior side of the rectum, even the size of organ deformation for the CTV was taken into account when estimating the blurred dose, resulting in a blurred dose that would be lower than the planned dose. In that case, higher dose constraints would be necessary, which could result in an inhomogeneous dose distribution in the CTV. To address this, we scaled up the dose constraint by 5% at the edge of the CTV. If a higher dose constraint was necessary, for example greater than 5%, this could require an inhomogeneous beam intensity inside the CTV, which could result in an inhomogeneous dose distribution. It should, therefore, be noted that the dose constraints and amount of deformation in the organ may vary, so the EE approach should be individualized for each patient.

Among the dose-volume parameters and radiobiological indices, a significant difference in gEUD for the CTV as small as 0.3 Gy was observed between the EE and PM approaches. Although it might be difficult to explain this difference and the resulting difference in TCP, van Herk et al. [33] noted that, when the dose difference and TCP difference with sufficient sampling was within 1%, these numerical solutions had the same probability distribution and so the gEUD and TCP values for the EE approach would be comparable to those for the PM approach. The HI was significantly larger with the EE approach than with the PM approach. D_98%_ with the EE approach was significantly smaller than with the PM approach (*p* < 0.001, data not shown), but the other parameters, D_2%_ and D_50%_, did not differ significantly. As van Herk et al. [33] noted, the minimum dose of the CTV is the most sensitive to geometric errors. This may explain the difference in HI in this study, because the D_98%_ could be regarded as the minimum dose [22].

For the rectum and bladder, all parameters evaluated with the EE approach were significantly smaller than those with the PM approach. These results were consistent with those reported by Trofimov et al. [13] and indicate that the EE approach was superior to the conventional margin technique with regard to the dose to the CTV and normal tissues. The difference between our study and theirs was that the dosimetric effect for normal tissues was evaluated accommodating their intrafraction organ deformation. Because the dose interplay effect in the CTV can be small for prostate IMRT [34], further attention should be paid to the dose blurring that can occur for both the target and normal tissue. In the present study, dose blurring may have occurred at the interface between the posterior side of the CTV and the anterior side of the rectum, and between the superior side of the CTV and the inferior side of the bladder. Such regions would receive an underdose due to organ motion. When the EE approach is used, especially if the organ motion is smaller than expected, the high dose region created at the edge of the CTV would be stable; in other words, it is highly probable that this high dose could be delivered to the normal tissue.

## Conclusions

Intrafraction organ deformation can be taken into account by incorporating the probability density function into the treatment planning process to ensure a robust dose distribution. In this study, the edge-enhanced technique was compared with the PTV-margin technique with regard to dosimetric and radiobiological parameters. The edge-enhanced technique resulted in superior dose reduction for the normal tissue while maintaining the dose coverage for the CTV, as well as reducing monitor units and the number of segments. The spatial margin with respect to the internal organ motion might be replaced with the dosimetric margin used in the edge-enhanced technique. A view of the dosimetric margin will support the robust treatment plan.
